# A Case of Transient Central Hypothyroidism Coinciding With Reversible Cerebral Vasoconstriction Syndrome

**DOI:** 10.7759/cureus.100051

**Published:** 2025-12-25

**Authors:** Hitomi Meshino, Asuka Uto, Yuki Ishinoda, Kazushi Suzuki, Naoki Oshima

**Affiliations:** 1 Department of Endocrinology and Metabolism, National Defense Medical College, Tokorozawa, JPN; 2 Division of Neurology, National Defense Medical College, Tokorozawa, JPN; 3 Department of Nephrology, National Defense Medical College, Tokorozawa, JPN

**Keywords:** central hypothyroidism, cerebrovascular disease, endocrine dysfunction, pituitary ischemia, reversible cerebral vasoconstriction syndrome

## Abstract

Reversible cerebral vasoconstriction syndrome (RCVS) is characterized by reversible segmental constriction and dilation of the major cerebral arteries and is typically unrelated to endocrine dysfunction. Central hypothyroidism (CH), a rare form of hypothyroidism caused by insufficient thyroid-stimulating hormone secretion from the pituitary gland, is usually associated with pituitary pathology. Here, we report a case of transient CH coinciding with RCVS, which to our knowledge has not been previously described in the literature. A 45-year-old woman presented with recurrent cerebral infarction characterized by total aphasia, right hemiplegia, and conjugate deviation. During the recurrent episode, endocrinological evaluation revealed CH with a blunted thyroid-stimulating hormone response to thyrotropin-releasing hormone stimulation, while other pituitary hormones remained normal. She was treated with levothyroxine and verapamil, leading to gradual improvement in both thyroid function and neurological deficits. Follow-up imaging demonstrated resolution of vascular stenosis, and levothyroxine was discontinued once thyroid function normalized. We hypothesize that transient vasoconstriction of small pituitary arteries, undetectable on imaging, may have contributed to reversible pituitary dysfunction. This case cautiously indicates a possible association between RCVS and CH, suggesting that endocrine evaluation would be helpful when managing patients with RCVS.

## Introduction

Reversible cerebral vasoconstriction syndrome (RCVS) is characterized by segmental constriction and dilation of the major cerebral arteries [1-4]. Although the precise mechanism remains incompletely understood, known triggers of RCVS include drugs, catecholamine-secreting tumors, trauma, and head or neck surgeries [1,2].

RCVS is an underrecognized and frequently misdiagnosed condition, often confused with other cerebrovascular diseases such as subarachnoid hemorrhage and intracerebral hemorrhage; therefore, the precise prevalence of RCVS is unknown [1]. No gender or racial predilection has been observed [2,3].

RCVS manifests with a broad range of symptoms, ranging from mild paresis to severe seizures, depending on the affected lesions. Despite its acute onset and clinical presentation, RCVS's natural history is typically benign, with complete remission of cerebral vasoconstriction within 12 weeks [4].

RCVS symptoms are primarily attributed to transient cerebral ischemia, and most patients present with neurological abnormalities; however, an association between RCVS and endocrinological dysfunction has not been reported.

Central hypothyroidism (CH) is a rare form of hypothyroidism in which the pituitary gland fails to produce sufficient thyroid-stimulating hormone (TSH) despite normal thyroid gland function [5]. CH commonly occurs secondary to pituitary disorders such as inflammation, infarction, or tumors [5]. Classic symptoms include fatigue, edema, constipation, and irregular menstruation. To date, no reports have described CH occurring in association with RCVS.

Here, we report a case of transient CH coinciding with RCVS. During the clinical course, CH gradually improved in association with the remission, suggesting a potential pathophysiological correlation between these diseases.

## Case presentation

A 45-year-old woman was transferred to our hospital due to recurrent cerebral infarction. One month earlier, she had been admitted to another neurology ward presenting with total aphasia, right hemiplegia, and left conjugate deviation. She was diagnosed with acute atherosclerotic cerebral infarction and underwent acute-phase treatment. Her condition improved post-treatment, and she was discharged on antiplatelet therapy. Several weeks later, she experienced recurrent right hemiplegia and sought another hospital. Magnetic resonance imaging (MRI) revealed a new infarction of the left putamen and caudate nucleus, prompting referral to our hospital for further management.

Laboratory findings on admission are presented in Table 1. The evaluation showed no signs of coagulopathy, cerebritis, encephalopathy, or viral-related infarctions. Further careful investigation revealed a history of mild intermittent headaches and irregular menstruation characterized by prolonged and irregular menstrual cycles during her previous hospitalization. She did not report vasomotor symptoms such as hot flashes.

**Table 1:** Laboratory data on admission.

Laboratory Parameter	Value	Normal Range
TSH	0.09	0.61-4.68 μIU/mL
Free T3	2.53	2.48-4.14 pg/mL
Free T4	0.75	0.76-1.65 ng/dL
TPOAb	12	<16 IU/mL
TgAb	19	<28 IU/mL
TRAb(Ⅲ)	0.5	<2.0 IU/mL
BUN	10	8-20 mg/dL
Cr	0.48	0.44-0.78 mg/dL
LDL cholesterol	80	<140 mg/dL
Triglycerol	114	30-150 mg/dL
Antithrombin	105	80-130 %
Activated protein C	115	64-146 %
Protein S	96	60-127 %
Lupus anticoreglant	1.1	<1.3
White blood cells	6800	4500-7500/μL
Hemoglobin	9.2	11.3-15.2g/dL
Hematocrit	30.6	36-45%
Platelet	513000	130000-350000/μL
ESR	18	＜20min

An endocrinological assessment was conducted, revealing CH. A thyrotropin-releasing hormone (TRH) stimulation test demonstrated a blunted TSH response (Table 2), while other pituitary hormones remained normal. Treatment with levothyroxine replacement was initiated at a low dose of 12.5 μg/day because she did not exhibit overt symptoms of hypothyroidism, together with verapamil at the standard dose of 120 mg/day as therapy for RCVS. After initiation of levothyroxine, free T3 and free T4 levels gradually improved, accompanied by normalization of the menstrual cycle.

**Table 2:** Endocrine function tests for endocrinology assessment. Peak values are indicated with asterisks. FSH: Follicle-stimulating hormone; TSH: thyroid-stimulating hormone; TRH: thyrotropin-releasing hormone; LH: luteinizing hormone; LHRH: luteinizing hormone-releasing hormone

Basal level TSH	0.7
TRH stimulation test	
TSH (30 min)	2.03*
TSH (60min)	2
TSH (90min)	1.59
TSH (120min)	1.3
Basal level LH	1.58
LHRH stimulation test	
LH (30min)	19.05
LH (60min)	19.57*
LH (90min)	17.15
LH (120min)	14.36
Basal level FSH	5.82
LHRH stimulation test	
FSH (30min)	17.4
FSH (60min)	19.87*
FSH (90min)	19.71
FSH (120min)	19.55
Basal level ACTH	8.6
CRH stimulation test	
ACTH (30min)	9.9
ACTH (60min)	9.7
ACTH (90min)	10.4*
ACTH (120min)	3.7

**Figure 1:**
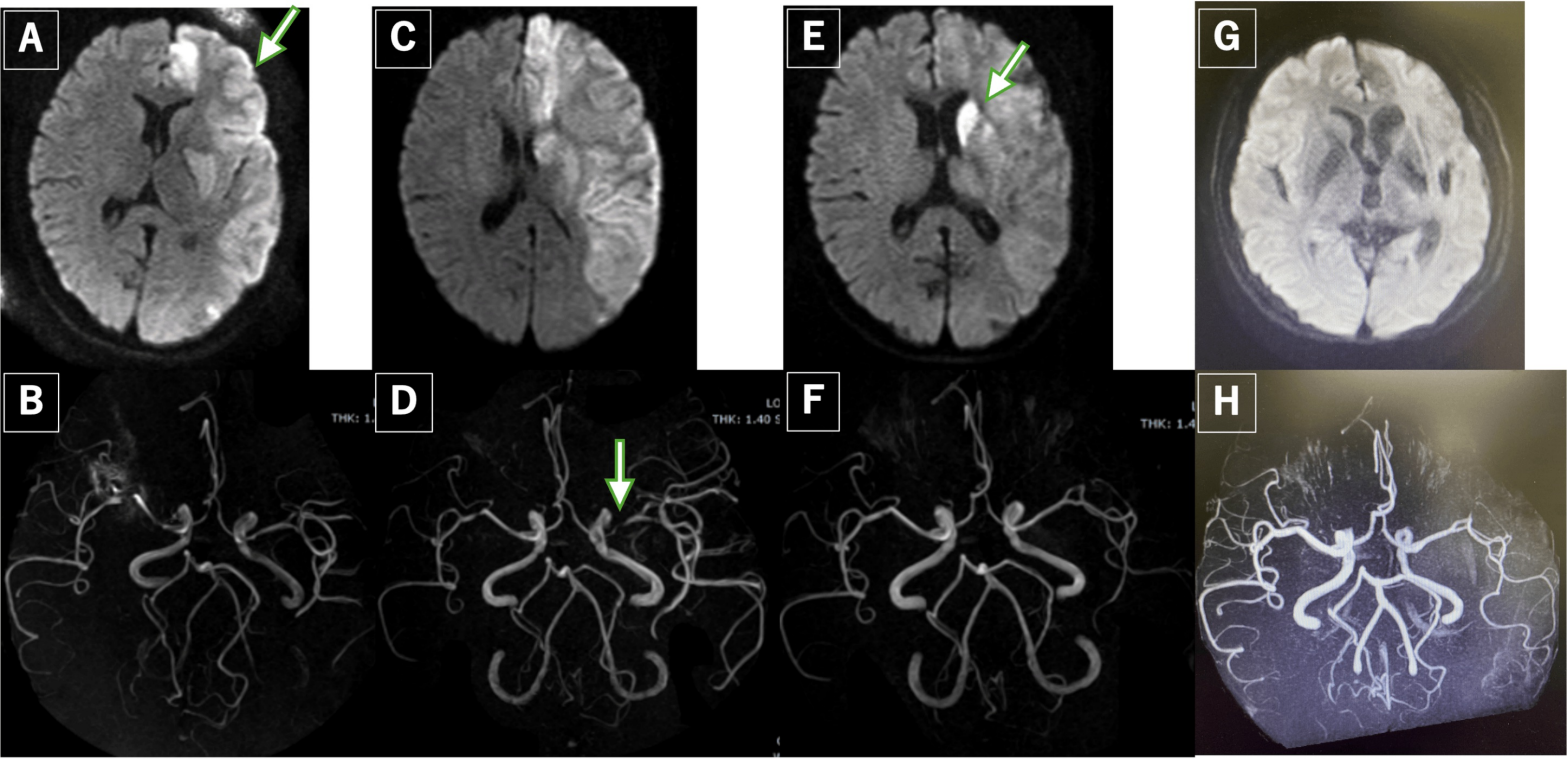
Brain MRI and MRA findings at presentation and follow-up. A: The initial diffusion-weighted imaging (DWI) obtained at the previous institution. The arrow indicates the extensive infarct lesions in the left anterior cerebral artery and middle cerebral artery territories. B: Magnetic resonance angiography (MRA) at the previous institution. C: DWI at our institution indicating the infarct of the left anterior cerebral artery and middle cerebral artery territories. D: MRA at our institution. The arrow indicates the vasospasm. E: DWI one month after initial presentation. New infarct lesions are identified in the left caudate nucleus and putamen (arrow). F: MRA one month after the initial presentation. G: A follow-up MRI performed six months later. H: MRA performed six months later showing resolution of the stenosis in the left anterior cerebral artery and middle cerebral artery.

Neurological symptoms, including aphasia after the initial episode and right hemiplegia after the recurrent episode, showed gradual improvement. Follow-up MRI at six months demonstrated resolution of stenosis in both the left anterior and middle cerebral arteries. Then, TSH levels gradually recovered, allowing for dose reduction and discontinuation of levothyroxine. Post-treatment, thyroid function remained stable within the normal limits (Figure 1).

## Discussion

Recently, a significant number of reports on RCVS have been published from various regions worldwide [1,2,4,6]. Although RCVS pathogenesis remains unclear, proposed mechanisms include sympathetic overactivity, endothelial dysfunction, and oxidative stress contributing to cerebral artery vasoconstriction [1,2]. The characteristic MRI finding in RCVS is reversible segmental dilatation and constriction of small vessels (the ‘strings of beads’ appearance), which typically resolves spontaneously within 20 weeks [2-4]. Thunderclap headache represents a major manifestation of RCVS, though it occurs independently of cerebral artery vasoconstriction. However, approximately 20% of patients may not experience thunderclap headache, as seen in our case [1,2].

RCVS diagnosis is established clinically based on diagnostic criteria, which require exclusion of other cerebrovascular diseases, including subarachnoid hemorrhage, cerebrospinal fluid abnormalities, cerebral amyloid angiopathy, meningitis, and cerebral infarction [1,3,4].

First-line treatment for RCVS consists of calcium channel blockers, which may alleviate cerebral artery spasms [6]. Adjunctive minor tranquilizers can be used for symptomatic management. However, serotonin reuptake inhibitor antidepressants are contraindicated due to their potential to exacerbate vasoconstriction [4].

The pituitary gland, often termed the “master gland”, regulates hormone secretion from the thyroid, gonadotropic, somatotropic, and corticotropic axes [5]. It receives blood supply via the superior and inferior hypophyseal arteries, which branch from the internal carotid arteries. Due to their small caliber, these arteries are typically undetectable on conventional tomographic examination. Therefore, impaired vascular perfusion may theoretically disrupt pituitary function, leading to systemic endocrine disturbances; however, no association between hemodynamic abnormalities and endocrine disorders has been reported [3,5,7]. Additionally, a retrospective study of 67 patients with RCVS found no patients with hypothyroidism [3]. Furthermore, in a cohort study of the general population in Denmark, 685 of 20,272,080 patients presented with hypothyroidism, but none of whom had RCVS [7].

One possible differential diagnosis for thyroid dysfunction in critically ill patients is euthyroid sick syndrome (ESS). However, ESS is typically characterized by decreased free T3 levels with normal or low-normal free T4 and suppressed or normal TSH, without a blunted TSH response to thyrotropin-releasing hormone. In our patient, the endocrinological evaluation demonstrated an impaired TSH response on TRH stimulation, supporting CH rather than ESS. Moreover, thyroid hormone replacement resulted in biochemical improvement, which would not be expected in transient ESS.

Sheehan syndrome, a well-characterized cerebrovascular disorder associated with endocrine dysfunction, also termed postpartum pituitary necrosis, typically results in panhypopituitarism or selective pituitary insufficiency [8]. Its pathogenesis involves severe postpartum hemorrhage-induced ischemia, leading to permanent hypophyseal hormone deficiency. In contrast, RCVS induces transient cerebrovascular stenosis, which may provoke reversible endocrine disturbance.

**Figure 2:**
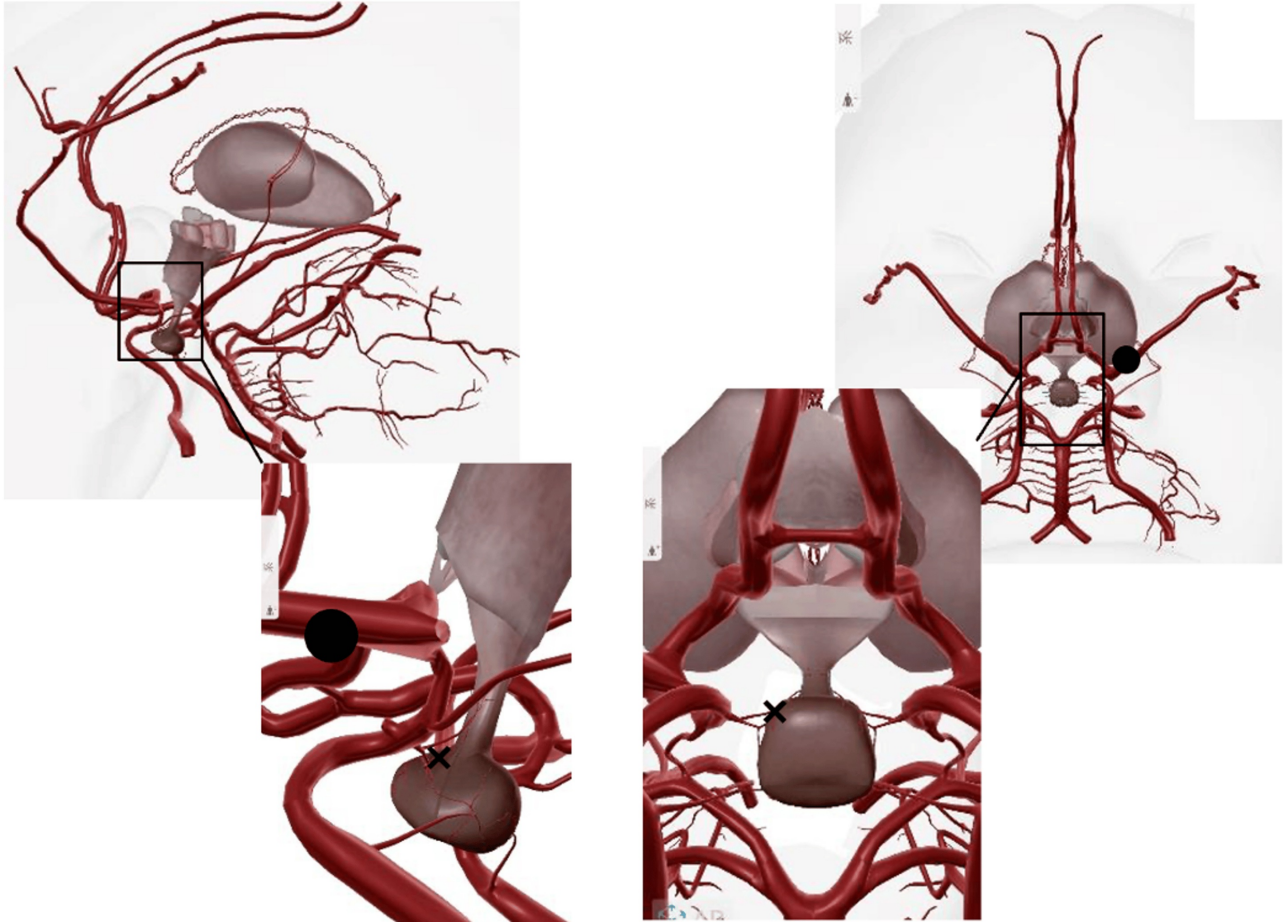
Schematic illustration of possible vascular involvement in this case. In this case, vasospasm was observed in the left anterior cerebral artery and middle cerebral artery territories.(⚫︎) The superior and inferior pituitary arteries are extremely thin and difficult to identify on imaging. （×） It cannot be ruled out that cerebral vasospasm occurred in the pituitary arteries or their branches during the process leading to this case of central hypothyroidism. Adapted with permission from the Human Anatomy Atlas (Visible Body) [9].

In our patient, MRI demonstrated stenosis of the left anterior and middle cerebral arteries. Although the location of the observed infarction itself could not directly explain impaired perfusion of the hypothalamic-pituitary axis, transient vasoconstriction of the hypophyseal arteries or their peripheral branches, below the spatial resolution of MRI, remains a plausible mechanism. Hypothyroidism and associated symptoms resolved concurrently with RCVS improvement, supporting a pathophysiological association between these conditions (Figure 2) [1,2,3,6].

## Conclusions

To our knowledge, this represents the first reported case of transient CH associated with RCVS. The parallel recovery of thyroid function and cerebrovascular abnormalities suggests a possible pathophysiological link between pituitary ischemia and RCVS. This case emphasizes that endocrine dysfunction, particularly CH, should be considered during the clinical course of RCVS. Early recognition and appropriate hormonal evaluation may contribute to accurate diagnosis and timely treatment. Further studies are warranted to clarify the underlying mechanisms and determine whether endocrine assessment should be incorporated into the routine evaluation of RCVS.
